# Implementing Psychosocial and Environmental Practices for Persons With Dementia in Assisted Living

**DOI:** 10.1093/geroni/igaa057.2352

**Published:** 2020-12-16

**Authors:** Lindsay Schwartz, Sheryl Zimmerman, Christopher Wretman, Lindsay Prizer, Philip Sloane

**Affiliations:** 1 American Health Care Association/ National Center for Assisted Living, Pittsboro, North Carolina, United States; 2 University of North Carolina at Chapel Hill, Chapel Hill, North Carolina, United States; 3 Emory University School of Medicine, Atlanta, Georgia, United States

## Abstract

Assisted living (AL) provides care for a large proportion of residents with dementia. Coincident with the increased focus on reducing off-label use of antipsychotics for people with dementia, providers are encouraged to turn to non-pharmacological practices to address behavioral expressions. This analysis used data from 250 AL communities in seven states, and examined familiarity, use, and practicality of twelve evidence-based practices, including music, pets, and social contact. Although a high percentage of staff reported familiarity and use of some of the practices, interviews with staff indicated that administration was not always consistent with evidence on implementation. Familiarity, use and practicality were associated with AL communities that had more residents with dementia, training on antipsychotics and non-pharmacological practices, policies on gradual dose reduction of psychotropics, and leadership that supported use of practices (p<.05 to <.001). Opportunities and barriers to implementation of these practices will be discussed.

